# Enhancement of cisplatin sensitivity in lewis lung carcinoma by liposome-mediated delivery of a survivin mutant

**DOI:** 10.1186/1756-9966-29-46

**Published:** 2010-05-12

**Authors:** Dan-Dan Yu, Chun-Ting Wang, Hua-Shan Shi, Zhi-Yong Li, Li Pan, Qing-Zhong Yuan, Fei Leng, Yuan Wen, Xiang Chen, Yu-Quan Wei

**Affiliations:** 1 State Key Laboratory of Biotherapy and Cancer Center, West China Hospital, West China Medical School, Sichuan University, Keyuan Road 4, Chengdu, Sichuan, China; 2 Department of tumor Oncology, Henan People's Armed Police Corps Hospital, China

## Abstract

**Background:**

A high concentration of cisplatin (CDDP) induces apoptosis in many tumor cell lines. CDDP has been administered by infusion to avoid severe toxicity. Recently, it has been reported that changes in survivin expression or function may lead to tumor sensitization to chemical and physical agents. The aim of this study was to determine whether a dominant-negative mouse survivin mutant could enhance the anti-tumor activity of CDDP.

**Methods:**

A plasmid encoding the phosphorylation-defective dominant-negative mouse survivin threonine 34→alanine mutant (survivin T34A) complexed to a DOTAP-chol liposome (Lip-mS) was administered with or without CDDP in Lewis Lung Carcinoma (LLC) cells and in mice bearing LLC tumors, and the effects on apoptosis, tumor growth and angiogenesis were assessed. Data were analyzed using one-way analysis of variance(ANOVA), and a value of *P *< 0.05 was considered to be statistically significant.

**Results:**

LLC cells treated with a combination of Lip-mS and CDDP displayed increased apoptosis compared with those treated with Lip-mS or CDDP alone. In mice bearing LLC tumors and treated with intravenous injections of Lip-mS and/or CDDP, combination treatment significantly reduced the mean tumor volume compared with either treatment alone. Moreover, the antitumor effect of Lip-mS combined with CDDP was greater than their anticipated additive effects.

**Conclusion:**

These data suggest that the dominant-negative survivin mutant, survivin T34A, sensitized LLC cells to chemotherapy of CDDP. The synergistic antitumor activity of the combination treatment may in part result from an increase in the apoptosis of tumor cells, inhibition of tumor angiogenesis and induction of a tumor-protective immune response.

## Background

There is a great deal of evidence that cisplatin (cis-diammine dichloroplatinum (II); CDDP) induces apoptosis in many tumor cell types. In the clinic, determining the greatest anti-tumoral efficiency using the lowest possible dose is a very difficult problem. Genetic therapy is considered to have enormous potential for resolving this issue.

A novel member of the inhibitor of apoptosis protein family (IAP), designated survivin [1], was recently identified by hybridization screening of human genomic libraries with the complementary DNA (cDNA) of a factor Xa receptor, effector cell protease receptor 1[2]. Unlike all other IAPs, survivin is expressed during development and by common human cancers, but is undetectable or detected at extremely low levels in normal adult tissues[1]. Survivin therefore has become an attractive target for novel anticancer interventional agents[3]. In recent years, considerable effort has been expended towards counteracting survivin, including development of antisense oligonucleotides[4], hammerhead ribozymes[5], RNA interference[6,7], cancer vaccines[8] and dominant-negative mutants[9]. Several preclinical studies have already demonstrated that down-regulation of survivin expression or function could inhibit tumor growth, increase spontaneous and induced apoptosis and sensitize tumor cells to anticancer agents.

Phosphorylation of survivin at Thr 34 by the cyclin-dependent kinase cdc2 is believed to promote physical interaction between survivin and caspase-9, resulting in caspase-9 inhibition to reduce apoptosis[10]. It was reported that the survivin mutant Thr34→Ala (survivin T34A) could abolish a phosphorylation site for cdc2-cyclin B1 and prevent survivin binding to activated caspase-9[11]. This reduced tumor cell proliferative potential and led to caspase-dependent apoptosis in melanoma cell lines[9]. It also increased the apoptosis of tumor cells, inhibited tumor angiogenesis and induced a tumor-protective immune response [11]. It was found that greater efficiency was attained in suppression of murine breast cancer by using a plasmid encoding the phosphorylation-defective mouse survivin T34A mutant complexed to DOTAP-chol liposomes (Lip-mS) [11]. As a result, the present study was designed to determine whether Lip-mS could enhance the antitumor activity of CDDP chemotherapy and to explore the possible mechanisms of interaction between survivin targeting-agents and chemotherapy.

## Methods

### Cell lines and culture conditions

The Lewis Lung Carcinoma (LLC) cell line of C57BL/6 mouse origin was purchased from the American Type Culture Collection (ATCC, Rockville, MD), cultured in DMEM (Gibco BRL, Grand Island, N.Y.) supplemented with 10% heat-inactivated fetal bovine serum (FBS), and maintained in a humidified incubator at 37°C in a 5% CO_2 _atmosphere.

### Plasmid DNA preparation

The recombinant plasmid encoding the phosphorylation-defective mouse survivin threonine 34→alanine mutant (pORF9-msurvivinT34A, mS) and pORF9-mcs (null plasmid) were each purchased from InvivoGen Corporation (San Diego, CA, USA) and confirmed by restriction endonuclease analysis, PCR and DNA sequence analysis. The plasmid was prepared using the Endofree Plasmid Giga kit (Qiagen, Chatsworth, CA). Endotoxin levels of the prepared plasmid DNA were determined by Tachypleus Amebocyte Lysate (TAL). No genomic DNA, small DNA fragments, or RNA were detected in the plasmid DNA and the OD260/280 ratios of the DNA were between 1.8 and 2.0. The DNA was dissolved in sterile endotoxin-free water and stored at -20°C until use.

### Preparation of DOTAP-chol liposome/plasmid DNA

DOTAP was purchased from Avanti Polar Lipids (Alabaster, AL) and highly purified cholesterol (Chol) was purchased from Sigma (St. Louis, MO). DOTAP-chol liposomes were prepared using the procedure described previously[11]. DNA:liposome mixtures were also prepared in accordance with a previously-described method [12]. Briefly, DOTAP-chol (20 mM) and plasmid DNA stock solution diluted in 5% dextrose in water (D5W) were mixed in equal volumes to give a final concentration of 4 mM DOTAP-chol, i.e., 150 μg DNA in 300 μL final volume (ratio, 1:2.6). These reagents were diluted and mixed at room temperature. The DNA solution was added to DOTAP-chol liposomes and rapidly mixed by pipetting up and down twice with the pipette tip. The DNA:liposome mixture thus prepared was precipitate-free and used for all the in vivo experiments. The size of the DNA fragments in the DNA:liposome mixture was determined to be in the range of 300-325 nm.

### Flow cytometric analysis

LLC cells were seeded in a 6-well plate and incubated for 24 h, then treated with normal saline (NS), CDDP, Lip-null, Lip-mS, or Lip-mS+CDDP (DNA at 1 μg/mL and CDDP at 4 μg/mL). Forty-eight hours later, the cells were washed with PBS and resuspended in propidium iodide/RNase A solution (0.5 mL), incubated at 37°C for 30 min and analyzed by flow cytometry.

### Animal studies

Studies involving whole mice were approved by the Institute's Animal Care and Use Committee. Female C57BL/6 mice of 6 to 8 weeks old were purchased from the experimental animal center of Sichuan University (Chengdu, Sichuan Province, China) and challenged subcutaneously (s.c.) with LLC cells (5 × 10^5 ^cells in 50 μL PBS) in the right flank. Mice were randomly divided into 4 groups (8 mice per group) and treated with NS, Lip-mS, CDDP or Lip-mS + CDDP until the tumors had mean diameter of 3 mm. Lip-mS was injected into mice via the tail vein at 5 μg per day once daily for 10 days (days 0 to 9) and CDDP (made in the Qilu Shandong Medical Factory) was injected into mice via the tail vein at 1 mg/kg per week (days 1, 8). Tumor size was determined by caliper measurement of the largest and perpendicular diameters every two days. Tumor volume was calculated according to the formula V = 0.52ab^2 ^(a is the largest superficial diameter and b is the smallest superficial diameter).

### Protein extraction and Western blot analysis

Tumor tissue samples were ground into powder under liquid nitrogen by milling in mortar, and lysed in RIPA lysis buffer (50 mM Tris-HCl (pH 7.4), 0.25% sodium deoxycholate, 150 mM NaCl, 1% nonidet P-40 (NP-40), 1 mM EDTA, 1 mM NaF, 1 mM Na3V4, 1 mM phenylmethylsulfonyl fluoride). After being quantifided by Bradford assay, lysates were subjected to 12% SDS-PAGE (sodium dodecyl sulfate polyacrylamide gel) electrophoresis, electroblotted with Sartoblot onto a PVDF membrane (Millipore, Bedford, MA) for 1 hr at 100 V, and then membrane blots were blocked at 4°C in 5% non-fat dry milk, washed, and probed with rabbit anti-mouse Caspase 9 antibody (Abcam, Cambridge, United Kingdom) at 1:1000 and anti-actin antibody (Santa Cruz Biotechnology, Santa Cruz, CA) at 1:100. The blots were labeled with horseradish peroxidase-conjugated secondary antibody and visualized by chemiluminescence detection.

### Terminal deoxynucleotidyl transferase-mediated dUTP nick end-labeling (TUNEL) assay

The mice were sacrificed by cervical dislocation on day 16 after the initiation of Lip-mS administration. Primary tumors were excised, fixed in 10% neutral-buffered formalin solution and embedded in paraffin. Contiguous 3-5 μm sections were mounted. In order to highlight the cells that were undergoing apoptosis, unstained sections mounted in silanized slides were subjected to fluorescent *in situ *TUNEL assay using an *in situ *apoptotic cell detection kit (Promega, Madison WI, USA), according to the manufacturer's protocol. Representative images were taken under a light microscope (×200) in randomly-selected fields.

### CD31 immunohistochemical evaluation

Immunohistochemistal analyses of microvessel formation were performed with goat anti-mouse CD31 antibody (Santa Cruz Biotechnology, Santa Cruz, CA) using the labeled streptavidin-biotin method. Briefly, sections were deparaffinized in xylol and rehydrated in a graded alcohol series. Antigen retrieval was carried out by autoclaving sections in retrieval buffer (10 mM EDTA citrate buffer, pH 6.0) for 3 min in saturated steam after up-pressure gaining (126, 1.6 bars, 23 psi). Endogenous peroxidase activity was blocked by incubation in 3% hydrogen peroxide at room temperature in the dark for 20 min. Non-specific binding of reagents was quenched by incubation of sections for 20 min in 5% normal rabbit serum. Sections were then incubated with goat anti-mouse CD31 (dilution 1/200) antibody overnight at 4°C, followed by incubation with biotinylated rabbit antigoat IgG, and then streptavidin-biotin-horseradish peroxidase complex at 37°C for 1 h. A negative control was included with each run by substituting the primary antibody with non-immune rabbit serum. Cellular nuclei were counterstained with ameliorative Gill's hematoxylin. Representative images were taken under a light microscope (×400) in randomly-selected fields.

### Statistical analysis

Statistical analysis of the differences in tumor volume, percent apoptosis and microvessel density were performed using one-way analysis of variance(ANOVA). A value of *P *< 0.05 was considered to be statistically significant.

## Results

### Enhancement of the anti-tumor effect of CDDP in vitro

**Figure 1:**
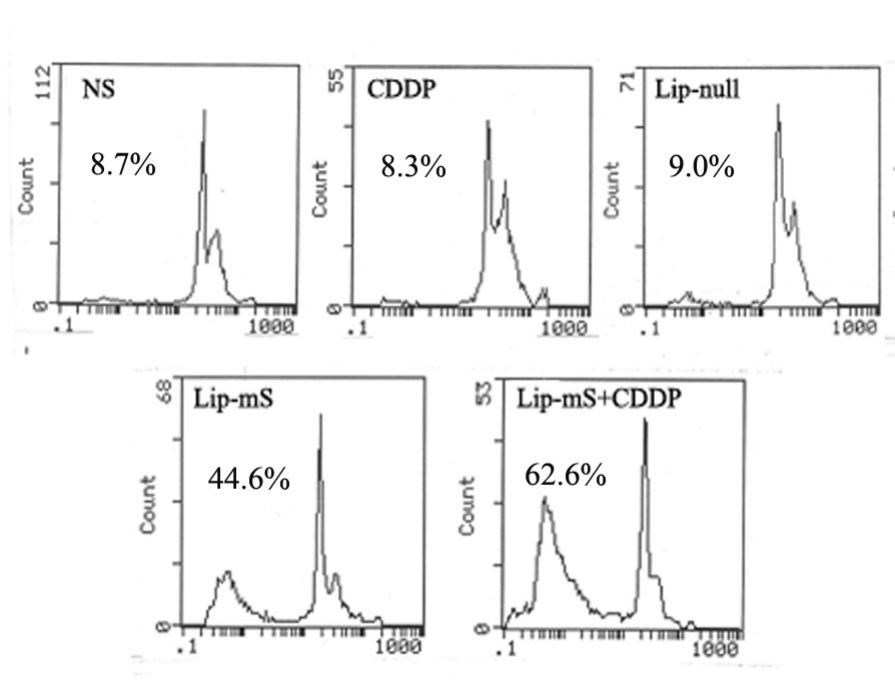
**Induction of apoptosis in LLC cells by treatment with Lip-mS and CDDP**. LLC cells were treated with NS (a), CDDP (b), Lip-null(c), Lip-mS (d), or Lip-mS+CDDP (e). Flow cytometric analysis revealed the proportion of sub-G1 cells (apoptotic cells) to be 8.7% (a), 8.3% (b), 9.0%(c)44.6% (d), and 62.6% (e), respectively.

In order to test the combined effect of Lip-mS with CDDP in vitro, we treated LLC cells with NS, CDDP (4 μg/mL), Lip-null (DNA at 1 μg/mL), Lip-mS (DNA at 1 μg/mL) or Lip-mS + CDDP. Growth inhibition was analyzed by measuring cell viability with flow cytometric analysis to evaluate the effect of Lip-mS and CDDP on the induction of apoptosis in LLC cells. Lip-mS + CDDP treatment significantly increased the proportion (62.6%) of sub-G1 cells (apoptotic cells) compared with the other treatments (NS, 8.7%; CDDP, 8.3%; Lip-null,9.0%;Lip-mS, 44.6%) (Fig. 1). Moreover, the interactive in vitro anti-tumor effect of the combined treatment was greater than the expected additive effect. The expected combined apoptotic effect was defined as follows: expected combined effect = Lip-mS effect + CDDP effect - Lip-mS effect × CDDP effect. The Lip-mS and CDDP treatment can induce apoptosis 44.6% and 8.3% respectively, so the expected induction of apoptosis in the combined treatment should be 49.2%. However, the actual induction of apoptosis in the combined treatment is 62.6%, suggesting greater than additive treatment effect.

### Enhancement of the anti-tumor effects of CDDP in vivo

**Figure 2:**
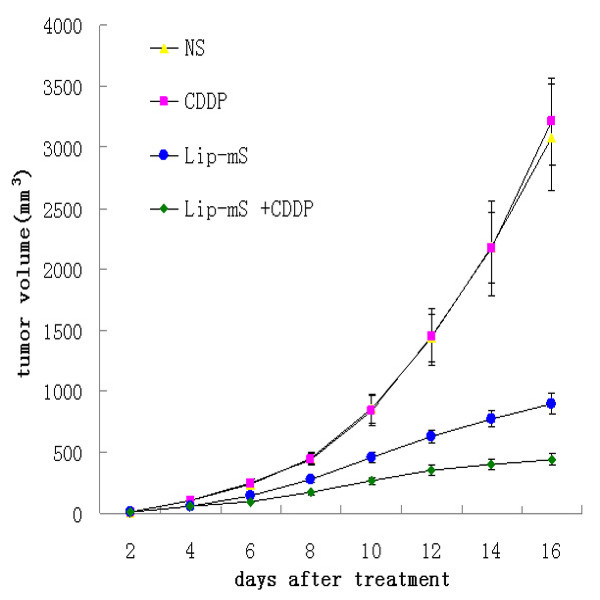
**Lip-mS enhanced the antitumor effects of CDDP in vivo**. Mice bearing LLC tumors were treated with NS, CDDP, Lip-mS or Lip-mS +CDDP. Combination treatment reduced the mean tumor volume on day 16 when compared with the Lip-mS or CDDP treatment group (*P *< 0.05).

**Figure 3:**
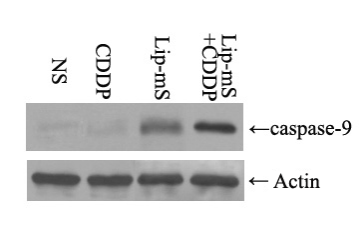
**Western blot analysis of caspase-9 expression in different groups**. Expression of caspase-9 was found in all groups. While no significant difference in the expression of anti-actin was found among them, caspase-9 was found to be expressed to a higher extent in Lip-mS + CDDP treatment groups as compared to NS, CDDP alone, Lip-mS alone treatment groups.

**Figure 4:**
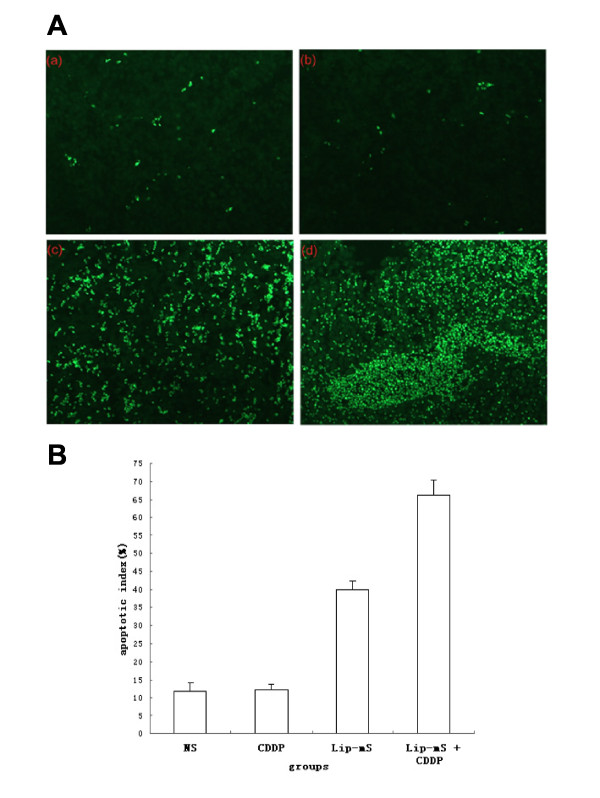
**Combination of Lip-mS and CDDP enhanced the induction of apoptosis in vivo**. Tissue sections from tumor-bearing mice treated with NS (a), CDDP (b), Lip-mS (c), or Lip-mS + CDDP (d) were stained with FITC-DUTP. Percent apoptosis was determined by counting the number of apoptotic cells and dividing by the total number of cells in the field (5 high power fields/slide). (A) Representative Field from each group. (B) Percent apoptosis in each group. Values were expressed as means ± SE. An apparent increase in the number of apoptotic cells was observed within tumors treated with a combination of Lip-mS and CDDP compared with the other treatments (*P *< 0.05).

**Figure 5:**
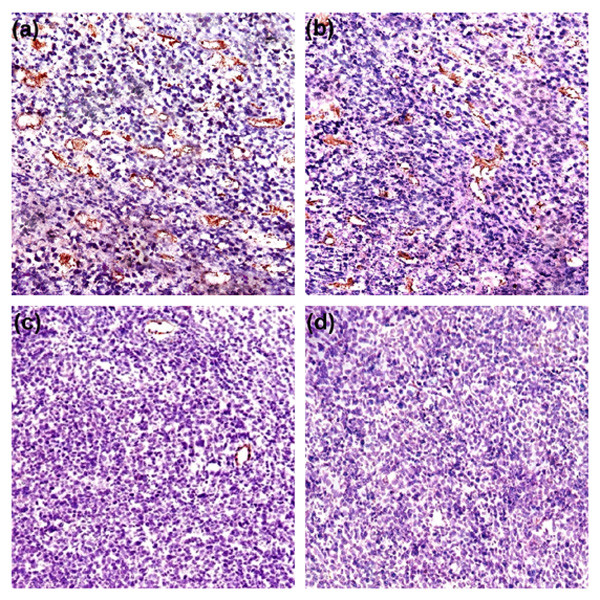
**Inhibition of intra-tumoral angiogenesis assayed by CD31 staining of microvessels**. Vascularization within tumors was detected by an antibody to CD31; representative images were taken under a light microscope (×400) in randomly-selected fields. Tumors of the NS (a) and CDDP (b) treatment groups demonstrated high microvessel density, while those of the Lip-mS (c) and Lip-mS + CDDP (d) treatment groups showed apparent inhibition of angiogenesis.

The anti-tumor effect of Lip-mS in combination with CDDP was assessed in mice bearing LLC tumors. The tumor growth curves demonstrated that, relative to NS or CDDP alone, Lip-mS resulted in effective suppression of tumor growth, while the combined treatment had a superior anti-tumor effect when compared with NS, Lip-mS or CDDP alone (*P *< 0.05) (Fig. 2). Moreover, the interactive anti-tumor effects of the combined treatment were also greater than their expected additive effects. On day 16 after the initiation of Lip-mS administration, the tumor inhibitory rate (TIR) of the CDDP group was zero. the TIR of Lip-mS alone was 71.1% and the combination treatment group was 85.9%. This suggests that combination treatment increased the inhibition, especially relative to CDDP (*P *< 0.05). In order to test by which possible mechanisms Lip-mS enhanced the anti-tumor effect of CDDP in vivo. The expression of caspase-9 in different treatment groups were detected by western blot. And tumor sections of each group were stained with TUNEL reagent and anti-CD31 antibody to evaluate the apoptotic rate and microvessel density. The details were described in Methods. Caspase-9 was found to be expressed to a higher extent in Lip-mS + CDDP treatment groups as compared to other groups(Fig. 3). And an apparent increase in the number of apoptotic cells was observed within the tumors treated with the combination of Lip-mS and CDDP compared with other treatments *(P *< 0.05) (Fig. 4). Tumors of the NS and CDDP-treated groups exhibited high microvessel density, while the density was reduced in the Lip-mS-alone and combination treatment groups (Fig. 5). These data suggest that Lip-mS can cause increased apoptosis of tumor cells and inhibition of tumor angiogenesis, which may play important roles in enhancement of the anti-tumor effects of chemotherapy in vivo.

## Discussion

Survivin has received much greater attention in recent years, thanks not only to its anti-apoptotic effects, but also its relation to chemoresistance. It was reported that survivin acts constitutively in a panel of tumor cells, and approaches designed to inhibit survivin expression or function may lead to tumor sensitization to chemical and physical agents [13]. Hence, the combination of genetic and chemotherapeutic approaches has been a topic of great interest.

CDDP is widely used for the treatment of a variety of human tumors such as lung cancer[14]. CDDP is a well-known DNA damaging agent, and it is currently thought that DNA platination is an essential first step in its cytotoxic activity[15]. However, continuous infusion or multiple administration of CDDP is an excellent regimen for cancer patients because of its adverse effects [16,17]. Therefore, approaches to improve the sensitivity to drug doses are a subject of intensive study in cancer care. Treatments combining genetic and chemotherapeutic approaches are a relatively new instrument in the fight against cancer.

Our study combining a Lip-mS genetic approach with CDDP significantly increased the anti-tumor effects of single chemotherapy. Moreover, the interactive anti-tumor effect of the combined treatment was greater than the expected additive effect. These data suggest that inhibition of survivin using a dominant-negative mutant, survivin T34A, can sensitize LLC cells to CDDP.

Reduction of apoptosis plays a very important role in tumor initiation, progression, and drug resistance. The major apoptosis-signaling pathways are the mitochondrial pathway and the death-receptor pathway. Several proteins that inhibit apoptosis have been identified, including the members of the bcl-2 family, such as bcl-2 and bcl-xL, and the IAPs. The anti-apoptotic proteins bcl-2 and bcl-xL block the apoptotic event of mitochondrial cytochrome *c *release into the cytosol, and have been shown to mainly inhibit these two above-mentioned pathways. The gene encoding the IAP survivin has been cloned, and the protein characterized [18]. Survivin is thought to be expressed in the G2/M phase of the cell cycle in a cell cycle-regulated manner, and to be associated with microtubule formation of the mitotic spindle[19,20].

As a member of the IAP family, survivin can block apoptosis triggered by a variety of apoptotic-stimulating factors. It can directly bind to and inhibit caspase-3 and caspase-7, which act at a common downstream part of the two major apoptotic pathways, and its overexpression in tumors has been implicated in resistance to a variety of apoptotic stimuli, including chemotherapy[17,20]. For this reason, the survivin antisense gene may facilitate both apoptotic pathways. Although survivin has long been considered a potential target for cancer therapy [18,19,21-25], the use of antisense cDNA and oligonucleotides to inhibit its expression has only recently been described [26,27]. Previous studies have shown that reduction of survivin expression achieved by antisense strategies results in apoptotic cell death and sensitization to anticancer drugs in several tumor cell lines [26,27]. These results suggest that survivin expression is likely important for cell survival or resistance to chemotherapy in carcinomas.

CDDP acts in the G2/M phase of the cell cycle. Previous studies have shown that an increase in chemosensitivity is negatively correlated with survivin expression and positively correlated with rates of apoptosis[28]. The results of the study by Kojima et al are consistent with expression of survivin in the G2/M phase[29]. These observations are consistent with an earlier finding [26] that interaction between survivin and microtubules of the mitotic spindle apparatus is necessary to prevent a default induction of apoptosis at the G2/M phase of the cell cycle. And it is reported that cisplatin induced caspase-9 activation and apoptosis in cisplatin-sensitive tumors[30]. Moreover, in a combination therapy experiment with CDDP, evidence was obtained that antisense-mediated downregulation of survivin can sensitize tumor cells to chemotherapy in vitro and in vivo [29].

## Conclusions

The survivin mutant had originally gained attention because it widely and specifically promoted apoptosis and enhanced chemotherapy, and its function and mechanism have been studied in various tumor types [9,11,12,29]. However, there are many aspects of its mechanisms that are still unclear. Our data suggest that the use of survivin T34A deserves further investigation as a useful approach to lung cancer therapy and in chemotherapy with CDDP. It was shown to down-regulate survivin expression and activity, to cause apoptosis in LLC cells, and to inhibit tumor growth. In addition, survivin T34A greatly enhances sensitivity to CDDP. These findings indicate the potential of this combination of a dominant-negative mutant--survivin T34A and administration of CDDP, or other chemotherapy, as a new therapeutic strategy for lung cancer.

## Competing interests

The authors declare that they have no competing interests.

## Authors' contributions

DDY carried out cell transfection, animal experiment, histologic analysis and drafted the manuscript. CTW participated in animal experiment, histologic analysis and helped to draft the manuscript. HSS and ZYL contributed to animal experiment. LP, FL, QZY and YW participated in plasmid DNA preparation. XC carried out Liposome preparation. YQW supervised experimental work and revised the manuscript. All authors read and approved the final manuscript.
